# Results of Levofloxacin Prophylaxis Timing in Autologous and Allogeneic Stem Cell Transplantation: A Retrospective Cohort Study

**DOI:** 10.7759/cureus.57598

**Published:** 2024-04-04

**Authors:** Sidika Gülkan Özkan, Seyedehtina Safaei, Ali Kimiaei, Yasemin Çınar, Meral Sönmezoğlu, Hasan Atilla Özkan

**Affiliations:** 1 Hematology, Bahçeşehir University, Istanbul, TUR; 2 Medical Sciences, Yeditepe University, Istanbul, TUR; 3 Infectious Diseases, Yeditepe University, Istanbul, TUR

**Keywords:** prophylactic antibiotics, fluoroquinolone, neutropenia, levofloxacin prophylaxis, hematopoietic stem cell transplantation, bacterial infections

## Abstract

Background

Despite preventive measures and varying antibiotic recommendations, bacterial infections continue to pose a significant threat to individuals undergoing hematopoietic stem cell transplantation (HSCT). Levofloxacin prophylaxis is commonly used, but the optimal timing for initiation is debated. This study aims to assess infection outcomes based on timing of levofloxacin prophylaxis (initiation at the first day of conditioning vs. after infusion of stem cells) in autologous and allogeneic HSCT patients.

Methods

We compared infectious episodes, responsible pathogens, and clinical outcomes based on the implementation of levofloxacin prophylaxis in patients receiving autologous or allogeneic HSCT procedures. This retrospective single-center study involved a review of the medical records of autologous and allogeneic HSCT patients treated at our adult stem cell transplantation unit between 2018 and 2020. The study included 23 patients who underwent autologous HSCT and 12 patients who underwent allogeneic HSCT. We compared the demographic data, febrile neutropenia, proven bacterial infections, and 30-day survival among the autologous and allogeneic transplant groups, including those who received oral levofloxacin 500 mg/day prophylaxis.

Results

Positive blood cultures (26.1% vs. 75%; p = 0.011), mean neutrophil engraftment (10.6±1.2 vs. 14.8±1.3; p<0.001), and mean platelet engraftment (11.2±1.1 vs. 15.4±3.2; p = 0.004) were all lower in autologous transplant patients versus their allogeneic counterparts. When each type of HSCT was evaluated within the same type, there were no observed differences in infection frequency, infection type, or 30-day mortality between the patient groups with different levofloxacin initiation times.

Conclusion

Healthcare professionals should choose the most appropriate timing for initiating levofloxacin prophylaxis based on individual patient factors and clinical circumstances while considering the cost-effectiveness implications. Further research with a larger sample size and prospective design is needed to support our findings.

## Introduction

Bacterial infections pose a significant health risk for individuals undergoing autologous and allogeneic hematopoietic stem cell transplantation (HSCT), leading to deaths and prolonged hospitalization. The presence of neutropenia, mucosal damage, and the use of a central catheter are the leading risk factors for bacterial infections in the early pre-engraftment phase [1]. Despite the current use of reduced-intensity conditioning regimens, prophylactic and preemptive antibiotic approaches, and improved supportive therapies, documented infections identified by positive blood cultures are still present in approximately 20-43% of cases [2-6]. Antibacterial prophylaxis with a fluoroquinolone (i.e., levofloxacin) to prevent bacterial infections should be strongly considered for adult HCT patients with anticipated neutropenic periods of seven days or more [7,8]. Antibacterial prophylaxis with fluoroquinolone has been shown in early randomized trials and meta-analyses to reduce all-cause mortality, infection-related mortality, fever, and infections in high-risk neutropenic patients, including allogeneic HCT recipients. However, the Centers for Disease Control and Prevention, the Infectious Diseases Society of America, and the American Society for Blood and Marrow Transplantation do not support this recommendation due to the emergence of resistant strains [9,10]. In 2017, the European Conference on Infections in Leukemia (ECIL) publications gave individual centers the discretion to decide on the use of prophylactic antibiotics, taking into account the balance of risks and benefits [11].

There are variable results in the literature regarding the efficacy of antibiotic prophylaxis in transplant recipients. Consequently, no standardized bacterial prophylaxis protocol exists for autologous and allogeneic HSCT. The decision-making process varies greatly from one center to another, mainly based on their individual experience and according to the causative agent seen in each center. Our study aimed to compare 30-day mortality, infection development rate, and causative pathogens in patients undergoing autologous and allogeneic HSCT at our center based on the timing of oral levofloxacin prophylaxis.

## Materials and methods

Patient population and treatment protocols

We retrospectively reviewed the medical records of patients undergoing autologous HSCT (AHSCT) and allogeneic HSCT (allo-HSCT) at the Yeditepe University Hospital's Adult Stem Cell Transplantation Unit between 2020 and 2022. A total of 23 patients (nine with multiple myeloma (MM) and 14 with non-Hodgkin's lymphoma (NHL)) underwent AHSCT, and 12 patients (10 with acute myeloid leukemia (AML), one with NHL, and one with acute lymphoblastic leukemia (ALL)) underwent allo-HSCT. As part of their conditioning regimen, the patients with MM received a dose of melphalan ranging from 140 to 200 mg/m^2^. The NHL patients undergoing AHSCT received a BEAM (bis-chloroethylnitrosourea, carmustine (BCNU)/carmustine, etoposide, cytarabine, and melphalan) conditioning regimen. Among the allo-HSCT patients, the majority (83.3%, n = 10) received a myeloablative conditioning regimen.

Data evaluation and definitions

In the data evaluation, it was observed that levofloxacin was started in some patients with the conditioning regimen and in some patients on the day of transplantation. Afterward, for AHSCT, the group started with the conditioning regimen was defined as O1, the group started on the day of transplantation was defined as O2, and for Allo-HSCT, the group started with the conditioning regimen was defined as A1, and the group started on the day of transplantation was defined as A2. Demographic data, the development of febrile neutropenia rate, the presence and type of bacterial infections, and 30-day survival were compared between the groups (O1 vs. O2, A1 vs. A2).

Statistical analysis

For statistical analysis, we utilized IBM SPSS Statistics for Windows, Version 20.0 (Released 2011; IBM Corp., Armonk, New York, United States). We represented numerical variables with a normal distribution as the mean ± standard deviation. While those without a normal distribution were presented as medians (min-max), categorical variables were represented using numbers and percentages. In order to compare categorical variables, we employed the Chi-square test, Yates' correction, and Fisher's exact test. For comparing the normal distribution of numerical variables between two groups, we used either the Student t-test or the Mann-Whitney U test. During our statistical analysis, a p-value of less than 0.05 was considered statistically significant.

## Results

The study population comprised 35 patients who underwent HSCT (mean age: 54.5±14.5 years; 18 females and 17 males). Among the patients, 25.7% (n = 9) had MM, 28.6% (n = 10) had AML and excess blast myelodysplastic syndrome (MDS), 42.9% (n = 15) had NHL, and 2.9% (n = 1) had ALL (chronic myeloid leukemia (CML) transformed). The AHSCT rate was 65.7% (n = 23), and the allo-HSCT rate was 34.3% (n = 12). Sixteen patients received one transplant (50.0%), while another 16 patients received two transplants (50.0%). Half of the patients received single-course treatment prior to transplantation. The distribution of mobilization regimens was as follows for the AHSCT patients: cyclophosphamide plus granulocyte-colony stimulating factor (G-CSF) in 39.1% (n = 9), etoposide plus G-CSF in 26.1% (n = 6), and mobilization with ongoing chemotherapy in 21.8% (n = 5). There were three patients (13%) with unknown mobilization regimens. Considering the whole patient group, 41.2% (n = 14) received a reduced intensity conditioning (RIC) regimen, and 58.8% (n = 20) received a myeloablative conditioning (MAC) regimen. In 62.9% (n = 22) of the patients, levofloxacin initiation occurred on the first day of conditioning, while the remaining patients started levofloxacin on the day of transplantation. The median graft volume was 5.9 kg (×106 CD34/kg) (range: 2.2-14 kg). Febrile neutropenia developed in 65.7% of the patients (n = 23), with a median onset day of 7 (range: 1-11). Gram-negative bacteria were detected in 22.9% (n = 8) of the patients, gram-positive bacteria in 17.1% (n = 6), and both gram-negative and gram-positive bacteria in 2.9% (n = 1). All patients received antifungal prophylaxis. One patient with a proven fungal infection was diagnosed with *Candida krusei*. The mean neutrophil engraftment was 11.9±2.3, and the mean platelet engraftment was 12.5±2.8. During the 30-day follow-up, one patient (2.9%) died. The median follow-up period was six months (range: 1-13), and no patient died after the 30-day follow-up (Table 1).

**Table 1:** Patient demographics and clinical findings. RIC: reduced intensity conditioning; MAC: myeloablative conditioning; AML: acute myeloid leukemia; MDS: myelodysplastic syndrome; CML: chronic myeloid leukemia; AHSCT: autologous hematopoietic stem cell transplantation; G-CSF: granulocyte colony-stimulating factor. Categorical variables were presented as numbers (%). Numerical variables were presented as mean ± standard deviation or median (min-max).

Variables	All patients (N=35)
Age (years), mean ± SD	54.5 ± 14.5
Gender, n (%)	
Female	18 (51.4)
Male	17 (48.6)
Diagnosis, n (%)	
Multiple myeloma	9 (25.7)
AML and MDS with excess blasts	10 (28.6)
Non-Hodgkin lymphoma	15 (42.9)
ALL (CML transformed)	1 (2.9)
Transplant type, n (%)	
Autologous	23 (65.7)
Allogeneic	12 (34.3)
Number of transplants, n (%)	
1	16 (50.0)
2	16 (50.0)
Mobilization regimen (AHSCT), n (%)	
Cyclophosphamide + G-CSF	9 (25.7)
Etoposide + G-CSF	6 (21.0)
Mobilization with ongoing chemotherapy	5 (20.0)
Unknown	15 (42.9)
Conditioning regimen, n (%)	
RIC	14 (41.2)
MAC	20 (58.8)
Initiation time of levofloxacin, n (%)	
With conditioning	22 (62.9)
At transplant day	13 (37.1)
Stem cell (×10^6^ CD34/kg)	5.9 (2.2-14)
Febrile neutropenia	23 (65.7)
Day	7 (1-11)
Bacterial growth in blood culture, n (%)	
Gram-negative	8 (22.9)
Gram-positive	6 (17.1)
Gram-negative and positive	1 (2.9)
No growth	20 (57.1)
Fungal infection prophylaxis, n (%)	35 (100.0)
Proven fungal infection, n (%)	
No	34 (97.1)
Yes	1 (2.9)
Neutrophil engraftment (days), mean ± SD	11.9 ± 2.3
Platelets engraftment (days), mean ± SD	12.5 ± 2.8
30th day survival, n (%)	
Alive	34 (97.1)
Dead	1 (2.9)
Overall survival, n (%)	
Alive	28 (96.6)
Dead	1 (3.4)
Follow-up time (months), median (range)	6 (1-13)

In patients who underwent AHSCT, the mean age was higher than that of those who underwent allogeneic transplantation (58.0±12.9 vs. 47.8±15.7; p = 0.048). Among the patients who underwent AHSCT, 39.1% (n = 9) had MM, and 60.9% (n = 14) had NHL. Among the patients who underwent Allo-HSCT, 83.3% (n = 10) had AML and excess blast MDS, 8.3% (n = 1) had NHL, and 8.3% (n = 1) had an ALL (CML-transformed) diagnosis (p<0.001). Bacterial growth in blood culture (26.1% vs. 75; p=0.011), mean neutrophil engraftment (10.6±1.2 vs. 14.8±1.3; p<0.001), and mean platelet engraftment (11.2±1.1 vs. 15.4±3.2; p=0.004) were lower in patients who underwent AHSCT compared to those who underwent Allo-HSCT. There were no significant differences between the transplantation types regarding other clinic findings (Table 2).

**Table 2:** Patient demographics and clinical findings by transplant type. RIC: reduced intensity conditioning; MAC: myeloablative conditioning; AML: acute myeloid leukemia; MDS: myelodysplastic syndrome; CML: chronic myeloid leukemia; AHSCT: autologous hematopoietic stem cell transplantation; G-CSF: granulocyte colony-stimulating factor. Categorical variables were shown as numbers (%). Numerical variables were expressed as mean ± standard deviation or median (min-max). p<0.05 indicates statistical significance.

Variables	Transplant type		p-value
	Autologous, n = 23	Allogeneic, n = 12	
Age (years), mean±SD	58.0±12.9	47.8±15.7	0.048
Gender, n (%)			
Female	9 (39.1)	9 (75.0)	0.075
Male	14 (60.9)	3 (25.0)	
Diagnosis, n (%)			
Multiple myeloma	9 (39.1)	0 (0)	
AML and MDS with excess blasts	0 (0)	10 (83.3)	
Non-Hodgkin lymphoma	14 (60.9)	1 (8.3)	
ALL (CML transformed)	0 (0)	1 (8.3)	
Number of transplants, n (%)			
1	12 (54.5)	4( 40.0)	0.703
2	10 (45.5)	6 (60.0)	
Mobilization regimen			
Cyclophosphamide + G-CSF	9 (39.1)	-	<0,001
Etoposide + G-CSF	6 (26.)	-	
Mobilization with ongoing chemotherapy	4 (17.4)	1 (8.3)	
Unknown	4 (17.4)	11 (91.7)	
Conditioning regime, n(%)			
RIC	12 (54.5)	2 (16.7)	0.075
MAC	10 (45.5)	10 (83.3)	
Initiation time of levofloxacin, n(%)			
With conditioning regimen	14 (60.9)	8 (66.7)	0.999
At transplant day	9 (39.1)	4 (33.3)	
Stem cell (×10^6^ CD34/kg)	6 (3.3-14)	5.9 (2.2-9.3)	0.534
Febrile neutropenia	13 (56.5)	10(83.3)	0.226
Development day	6 (4-9)	9(1-11)	0.208
Bacterial growth in blood culture, n(%)			
No growth	17 (73.9)	3 (25.0)	0.016
Growth	6 (26.1)	9 (75.0)	
Gram-negative	2 (8.7)	6 (50.0)	0.006
Gram-positive	3 (13.0)	3 (25.0)	
Gram-negative and gram-positive	1 (4.3)	-	
Fungal infection prophylaxis, n (%)	23 (100.0)	12 (100.0)	-
Proven fungal infection, n (%)			
No	22 (95.7)	12 (100.0)	0.999
Yes	1 (4.3)	0 (0)	
Neutrophil engraftment (days), mean±SD	10.6±1.2	14.8±1.3	<0.001
Platelets engraftment (days), mean±SD	11.2±1.1	15.4±3.2	0.004
30th day survival, n (%)			
Alive	23 (100.0)	11 (91.7)	0.737
Dead	0 (0)	1 (8.3)	
Overall survival, n(%)			
Alive	17 (100.0)	11 (91.7)	0.859
Dead	0 (0)	1 (8.3)	
Follow-up time (months), median (range)	6 (0-13)	6 (0-12)	0.817

No noteworthy differences existed in demographic and clinical findings between patients who were given a conditioning regimen and those who initiated the treatment on the day of transplantation. This observation applied to both autologous and allogeneic HSCT (Table 3).

**Table 3:** Distribution of demographic and clinical findings according to the initiation time of levofloxacin in transplantation types. RIC: reduced intensity conditioning; MAC: myeloablative conditioning; AML: acute myeloid leukemia; MDS: myelodysplastic syndrome; CML: chronic myeloid leukemia; AHSCT: autologous hematopoietic stem cell transplantation; G-CSF: granulocyte colony-stimulating factor. Categorical variables were shown as numbers (%). Numerical variables were expressed as mean ± standard deviation or median (min-max). p<0.05 indicates statistical significance.

Variables	Autologous		p-value	Allogeneic			p-value
	Levofloxacin starting time			Levofloxacin starting time			
	O1, n=14	O2, n=9		A1, n=8	A2, n=4		
Age (years), mean±SD	58.2±12.6	57.7±14.2	0.924	47.6±17.5	48.3±13.6		0.952
Gender, n (%)							
Female	7 (50.0)	2 (22.2)	0.371	6 (75.0)	3 (75.0)		0.999
Male	7 (50.0)	7 (77.8)		2 (25.0)	1 (25.0)		
Diagnosis, n (%)							
Multiple myeloma	6 (42.9)	3 (33.3)	0.985	-	-		-
AML and MDS with excess blasts	-	-		7 (87.5)	3 (75.0)		
NHL	8 (57.1)	6 (66.7)		1 (12.5)	-		
ALL (CML transformed)	-	-		-	1 (25.0)		
Number of transplants, n (%)							
1	6 (46.2)	6 (66.7)	0.607	1 (16.7)	3 (75.0)		0.236
2	7 (53.8)	3 (33.3)		5 (83.3)	1 (25.0)		
Mobilization regimen (AHSCT), n (%)							
Cyclophosphamide + G-CSF	6 (42.9)	3 (33.3)	0.700	-	-	0.999	
Etoposide + G-CSF	3 (21.4)	3 (33.3)		-	-		
Mobilization with the ongoing chemotherapy	3 (21.4)	1 (11.1)		1 (12.5)	-		
Unknown	2 (14.3)	2 (22.2)		7 (87.5)	4 (100)		
Conditioning regimen, n (%)							
RIC	6 (46.2)	6 (66.7)	0.607	1 (12.5)	1 (25.0)		0.999
MAC	7 (53.8)	3 (33.3)		7 (87.5)	3 (75.0)		
Stem cell (×10^6^ CD34/kg), median (range)	5.6 (3.3-14)	6.1 (4.4-9.8)	0.695	6.2 (2.2-9.3)	5 (3.9-6.7)		0.776
Febrile neutropenia, n (%)	8 (57.1)	5 (55.6)	0.999	7 (87.5)	3 (75.0)		0.990
Development day, median (range)	7 (4-9)	6 (5-6)	0.435	9 (1-11)	9 (3-11)		0.997
Bacterial growth in blood culture, n (%)							
Gram-negative	1 (7.1)	1 (11,1)	0.383	5 (62.5)	1 (25.0)		0.473
Gram-positive	3 (21.4)	0 (0)		2 (25.0)	1 (25.0)		
Gram-negative and gram-positive	-	1 (11.1)		-	-		
No growth	10 (71.4)	7 (77.8)		1 (12.5)	2 (50.0)		
Fungal infection prophylaxis, n (%)	14 (100.0)	9 (100.0)		8 (100.0)	4 (100.0)		
Proven fungal infection, n (%)							
No	13 (92.9)	9 (100.0)	0.999	8 (100.0)	4 (100.0)		
Yes	1 (7.1)	0 (0)		-	-		
Neutrophil engraftment (days), mean±SD	10.6±1.1	10.4±1.3	0.699	14.9±1.5	14.8±1		0.900
Platelet engraftment (days), mean±SD	11±1	11.3±1.2	0.511	15.7±2.9	15±4.4		0.790
30th day survival, n (%)							
Alive	14 (100.0)	9 (100.0)	-	7 (87.5)	4 (100.0)		0.999
Dead	-	-		1 (12.5)	-		
Overall survival, n (%)							
Alive	11 (100.0)	6 (100.0)	-	7 (87.5)	4 (100.0)		0.999
Dead	-	-		1 (12.5)	-		
Follow-up time (months), median (range)	5.5 (0-13)	6 (2-12)	0.868	6 (0-8)	8.5 (4-12)		0.283

## Discussion

Due to the immunosuppression, prolonged neutropenia, and use of central line access associated with HSCT, infections pose a substantial risk and contribute significantly to morbidity and mortality in stem cell recipients [1,12-14]. In this study, we aimed to assess these outcomes by analyzing the timing of levofloxacin prophylaxis initiation while concurrently evaluating the clinical outcomes and 30-day mortality rates among patients who experienced infection. No significant differences were detected among the transplantation types concerning other clinic findings. Furthermore, we discovered no differences in infection frequency, infection type, or 30-day mortality between patients undergoing autologous and allogeneic HSCT who received levofloxacin prophylaxis, regardless of whether it began during hospitalization or on the day of transplantation. This suggests that the timing of levofloxacin administration did not impact these specific outcomes for either transplantation group. Notably, there is a lack of specific studies in the literature addressing the optimal timing of levofloxacin administration in this context.

It is recommended in the literature to start bacterial prophylaxis during stem cell infusion and continue it until neutropenia resolves or empirical antibacterial therapy for febrile neutropenia is started [8]. While fluoroquinolone prophylaxis has been shown to be effective in reducing gram-negative infections in neutropenic patients, its benefits on mortality have not been consistently demonstrated [10,15-18] Furthermore, fluoroquinolone prophylaxis has been linked to a higher risk of fluoroquinolone-resistant gram-negative bacteria and *Staphylococcus* species, as well as the development of *Clostridium difficile*-associated diarrhea [19-21] In contrast, another study found that fluoroquinolone prophylaxis reduced the incidence of bacteremia after autologous hematopoietic cell transplantation without increasing the occurrence of *C. difficile* infections [22]. Furthermore, fluoroquinolone-resistant bacteremia has been linked to a higher mortality rate than fluoroquinolone-sensitive bacteremia [23]. Comparative studies in the literature, which have examined groups using different medications or compared medication usage with non-usage, found that levofloxacin prophylaxis can decrease the frequency of central line-associated infections but may increase the occurrence of infections caused by resistant strains [24]. No resistant bacteria or *Clostridium* infections were found in our study. However, since a group of patients who did not use levofloxacin was not included in this study, no comparison could be made in this respect. The risk of resistant organisms and secondary infections, such as *C. difficile* infection [9,10], contributes to the discontinuation of standardized recommendations for bacterial infection prophylaxis in stem cell transplantation and accounts for observed variation among transplantation centers, resulting in a diverse approach to prophylactic antibiotic use based on each center's experience and risk-benefit assessment.

According to a recent survey conducted within the Blood and Marrow Transplant Clinical Trials Network (BMT-CTN) centers, roughly 75% of the centers use fluoroquinolones as a preventive measure against bacterial infections (levofloxacin in 80% and ciprofloxacin in 20%). In contrast, 15% of the centers do not use bacterial prophylaxis. Around 70% of the centers adopt prophylaxis initiation on specific dates (55% before and approximately 15% after day 0), whereas the remaining centers initiate the treatment on the occurrence of neutropenia [25]. During the period when these data were collected, levofloxacin was used in all patients in our center, but the time of initiation of treatment varied.

Our study yielded no significant variations in demographic or clinical findings between patients who were administered levofloxacin during hospitalization versus those who initiated it on the day of transplantation. Multiple factors may contribute to this outcome, which should be acknowledged. Implementing other preventive strategies, such as antifungal prophylaxis and infection control techniques, was crucial in reducing infections and lowering discrepancies among both groups. Moreover, regardless of when levofloxacin prophylaxis was initiated, its adequacy for reducing infections might have been comparable regardless of the timing of initiation. Differences in diagnoses, transplant types (autologous vs. allogeneic), conditioning regimens (myeloablative vs. reduced-intensity), and the random assignment of levofloxacin initiation timing may also contribute to variations in outcomes.

The limitations of this study include its retrospective design, small sample size, single-center nature, and a limited follow-up period of six months. Additionally, the relationship between levofloxacin prophylaxis and graft-versus-host disease (GVHD) has not been addressed. Lastly, omitting a comparison group that did not receive levofloxacin prophylaxis obstructs us from executing direct outcome comparisons between individuals who were on prophylaxis versus those who were not. Thus, future research initiatives should diligently address these limitations in order to foster a deeper understanding within this realm.

## Conclusions

Our study provides valuable insights into the timing of levofloxacin prophylaxis in autologous and allogeneic HSCT patients. Our findings suggest that the timing of levofloxacin initiation, whether during hospitalization or on the day of transplantation, may not significantly impact infection outcomes or mortality rates in this patient population. However, it's crucial to interpret these findings with caution due to the limitations of our study, including the small sample size and retrospective design. Healthcare professionals should continue to consider individual patient factors and clinical circumstances when determining prophylactic strategies, while remaining vigilant for emerging evidence in this area. Future research with larger sample sizes and prospective study designs is warranted to corroborate our findings and provide more robust evidence.

## References

[1] Sahin U, Toprak SK, Atilla PA, Atilla E, Demirer T (2016). An overview of infectious complications after allogeneic hematopoietic stem cell transplantation. J Infect Chemother.

[2] Meyer E, Beyersmann J, Bertz H (2007). Risk factor analysis of blood stream infection and pneumonia in neutropenic patients after peripheral blood stem-cell transplantation. Bone Marrow Transplant.

[3] Oliveira AL, de Souza M, Carvalho-Dias VM (2007). Epidemiology of bacteremia and factors associated with multi-drug-resistant gram-negative bacteremia in hematopoietic stem cell transplant recipients. Bone Marrow Transplant.

[4] Poutsiaka DD, Price LL, Ucuzian A, Chan GW, Miller KB, Snydman DR (2007). Blood stream infection after hematopoietic stem cell transplantation is associated with increased mortality. Bone Marrow Transplant.

[5] Mikulska M, Del Bono V, Raiola AM (2009). Blood stream infections in allogeneic hematopoietic stem cell transplant recipients: reemergence of gram-negative rods and increasing antibiotic resistance. Biol Blood Marrow Transplant.

[6] Hong J, Moon SM, Ahn HK (2013). Comparison of characteristics of bacterial bloodstream infection between adult patients with allogeneic and autologous hematopoietic stem cell transplantation. Biol Blood Marrow Transplant.

[7] Giampaolo B, Elio C, Claudio V (2007). Quinolone prophylaxis for bacterial infections in afebrile high risk neutropenic patients. Eur J Cancer.

[8] Tomblyn M, Chiller T, Einsele H (2009). Guidelines for preventing infectious complications among hematopoietic cell transplantation recipients: a global perspective. Biol Blood Marrow Transplant.

[9] (2000). Guidelines for preventing opportunistic infections among hematopoietic stem cell transplant recipients. MMWR Recomm Rep.

[10] Trifilio S, Verma A, Mehta J (2004). Antimicrobial prophylaxis in hematopoietic stem cell transplant recipients: heterogeneity of current clinical practice. Bone Marrow Transplant.

[11] Mikulska M, Averbuch D, Tissot F (2018). Fluoroquinolone prophylaxis in haematological cancer patients with neutropenia: ECIL critical appraisal of previous guidelines. J Infect.

[12] Jacobson S, Clemmons AB, Shah A (2016). Impact of fluoroquinolone prophylaxis on survival outcomes after hematopoietic stem cell transplantation: a single-center experience. Biol Blood Marrow Transplant.

[13] Neofytos D (2019). Antimicrobial prophylaxis and preemptive approaches for the prevention of infections in the stem cell transplant recipient, with analogies to the hematologic malignancy patient. Infect Dis Clin North Am.

[14] Busca A, Cinatti N, Gill J (2021). Management of invasive fungal infections in patients undergoing allogeneic hematopoietic stem cell transplantation: the Turin experience. Front Cell Infect Microbiol.

[15] Cruciani M, Rampazzo R, Malena M, Lazzarini L, Todeschini G, Messori A, Concia E (1996). Prophylaxis with fluoroquinolones for bacterial infections in neutropenic patients: a meta-analysis. Clin Infect Dis.

[16] van de Wetering MD, de Witte MA, Kremer LC, Offringa M, Scholten RJ, Caron HN (2005). Efficacy of oral prophylactic antibiotics in neutropenic afebrile oncology patients: a systematic review of randomised controlled trials. Eur J Cancer.

[17] Bucaneve G, Micozzi A, Menichetti F (2005). Levofloxacin to prevent bacterial infection in patients with cancer and neutropenia. N Engl J Med.

[18] Viscoli C (2007). Antibacterial prophylaxis in neutropenic patients. Int J Antimicrob Agents.

[19] Graffunder EM, Venezia RA (2002). Risk factors associated with nosocomial methicillin-resistant Staphylococcus aureus (MRSA) infection including previous use of antimicrobials. J Antimicrob Chemother.

[20] Craig M, Cumpston AD, Hobbs GR, Devetten MP, Sarwari AR, Ericson SG (2007). The clinical impact of antibacterial prophylaxis and cycling antibiotics for febrile neutropenia in a hematological malignancy and transplantation unit. Bone Marrow Transplant.

[21] Chong Y, Yakushiji H, Ito Y, Kamimura T (2011). Clinical impact of fluoroquinolone prophylaxis in neutropenic patients with hematological malignancies. Int J Infect Dis.

[22] Clemmons AB, Gandhi AS, Albrecht B, Jacobson S, Pantin J (2019). Impact of fluoroquinolone prophylaxis on infectious-related outcomes after hematopoietic cell transplantation. J Oncol Pharm Pract.

[23] Miles-Jay A, Butler-Wu S, Rowhani-Rahbar A, Pergam SA (2015). Incidence rate of fluoroquinolone-resistant gram-negative rod bacteremia among allogeneic hematopoietic cell transplantation patients during an era of levofloxacin prophylaxis. Biol Blood Marrow Transplant.

[24] Ziegler M, Landsburg D, Pegues D (2019). Fluoroquinolone prophylaxis is highly effective for the prevention of central line-associated bloodstream infections in autologous stem cell transplant patients. Biol Blood Marrow Transplant.

[25] Rashidi A, Wangjam T, Bhatt AS, Weisdorf DJ, Holtan SG (2018). Antibiotic practice patterns in hematopoietic cell transplantation: a survey of blood and marrow transplant clinical trials network centers. Am J Hematol.

